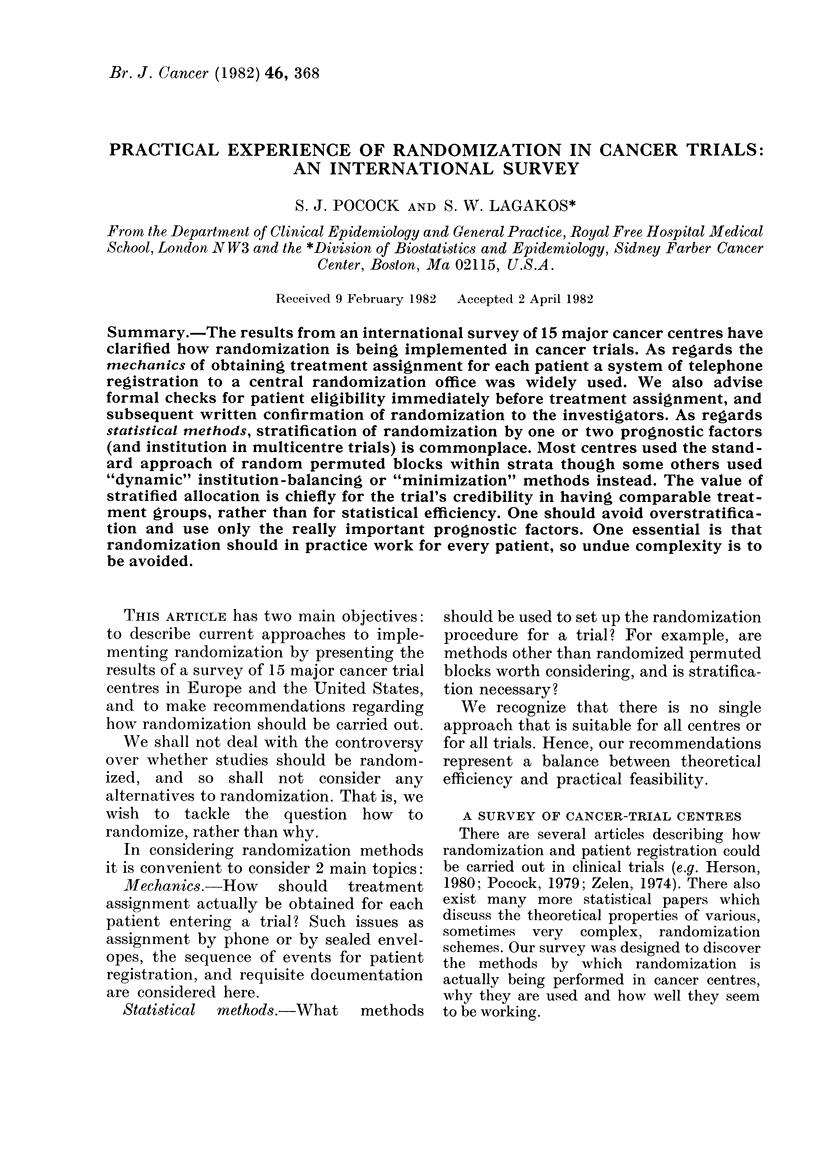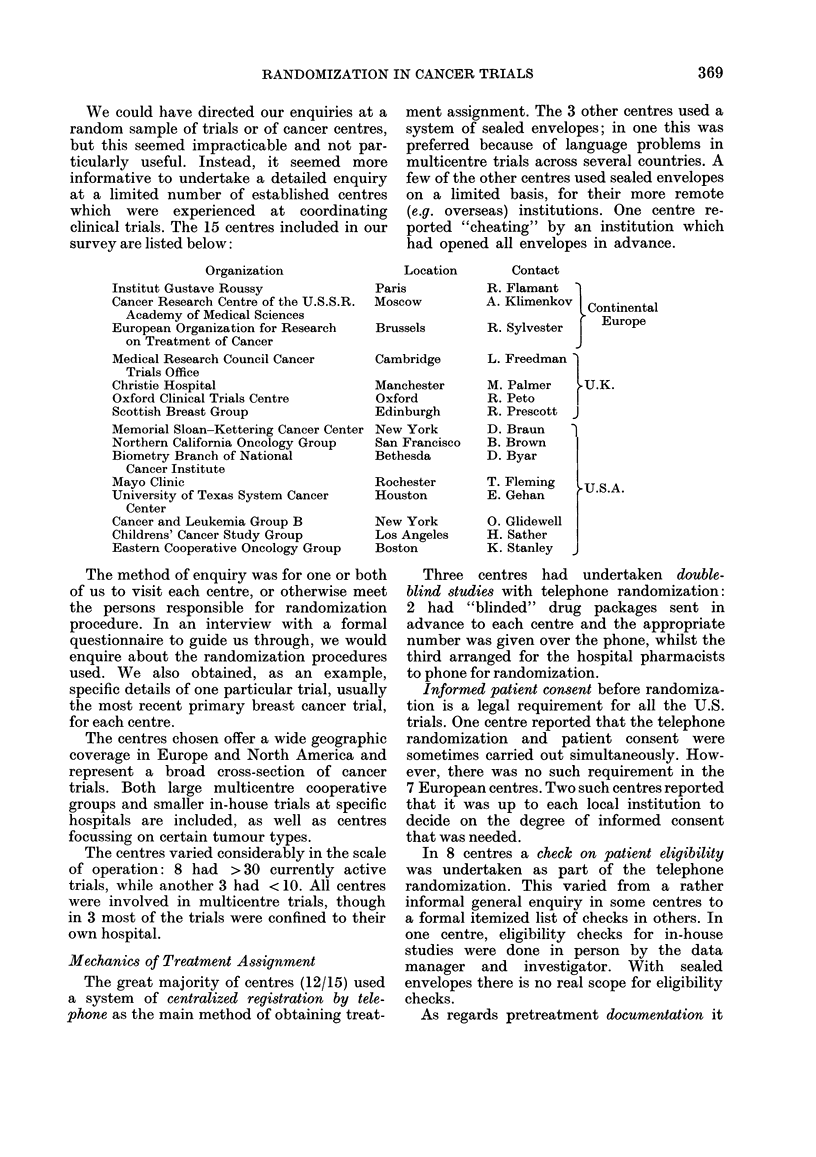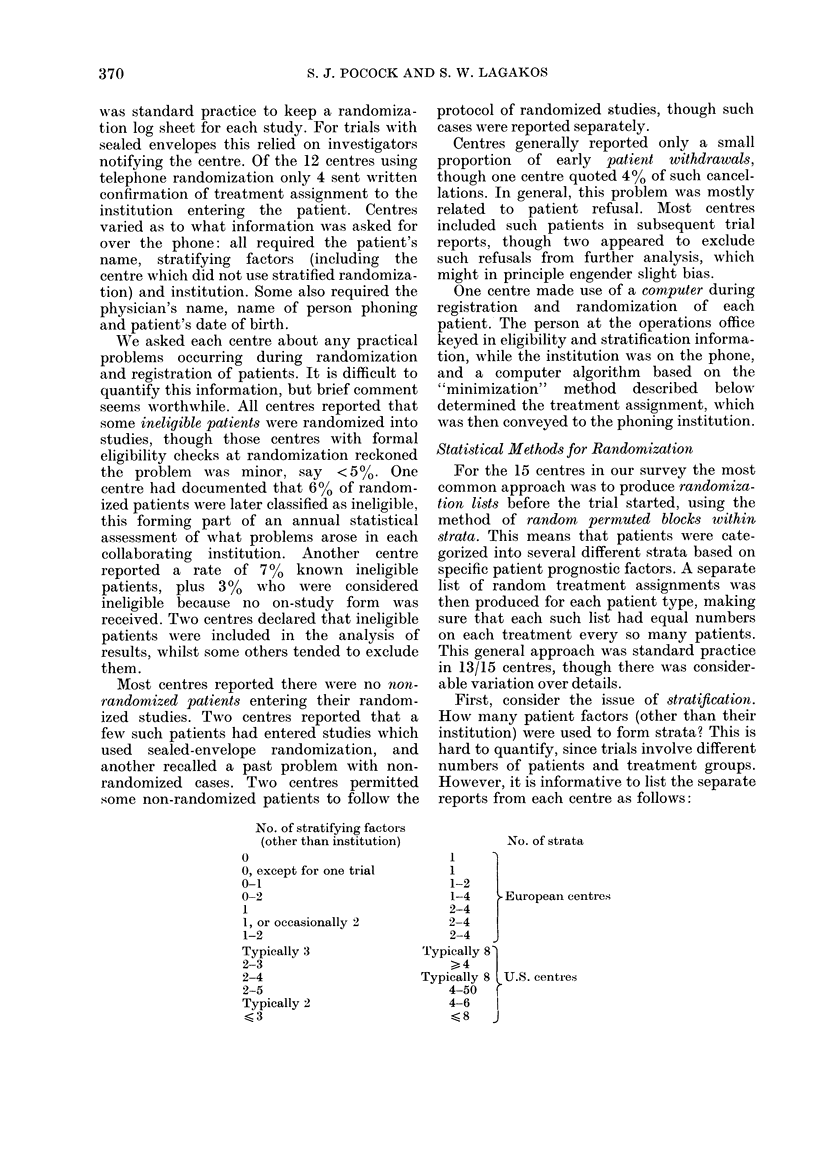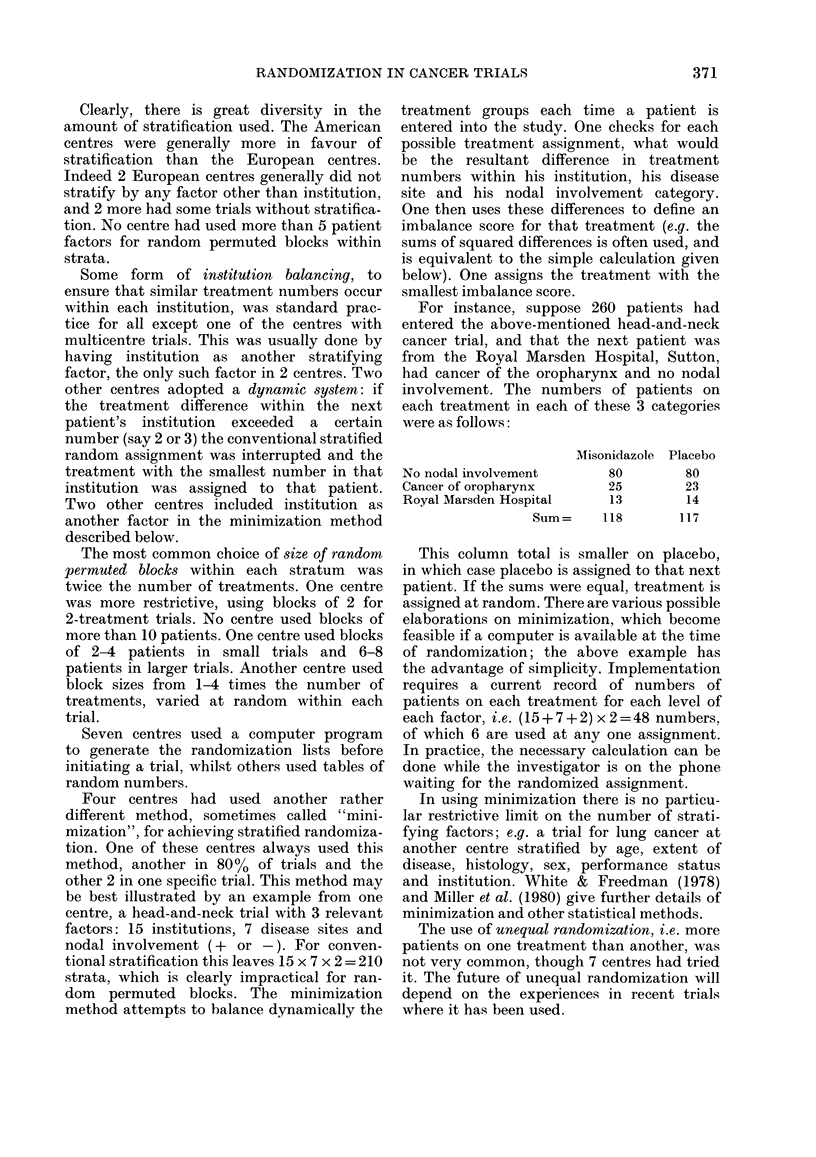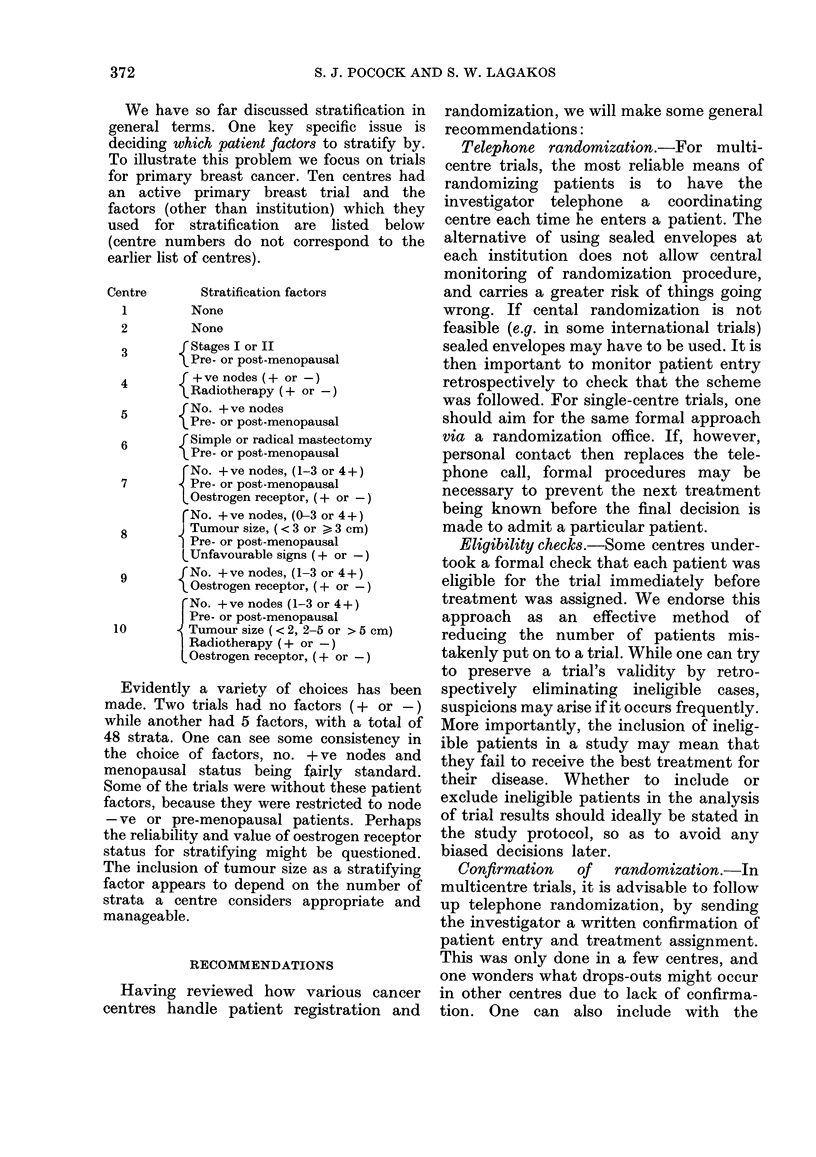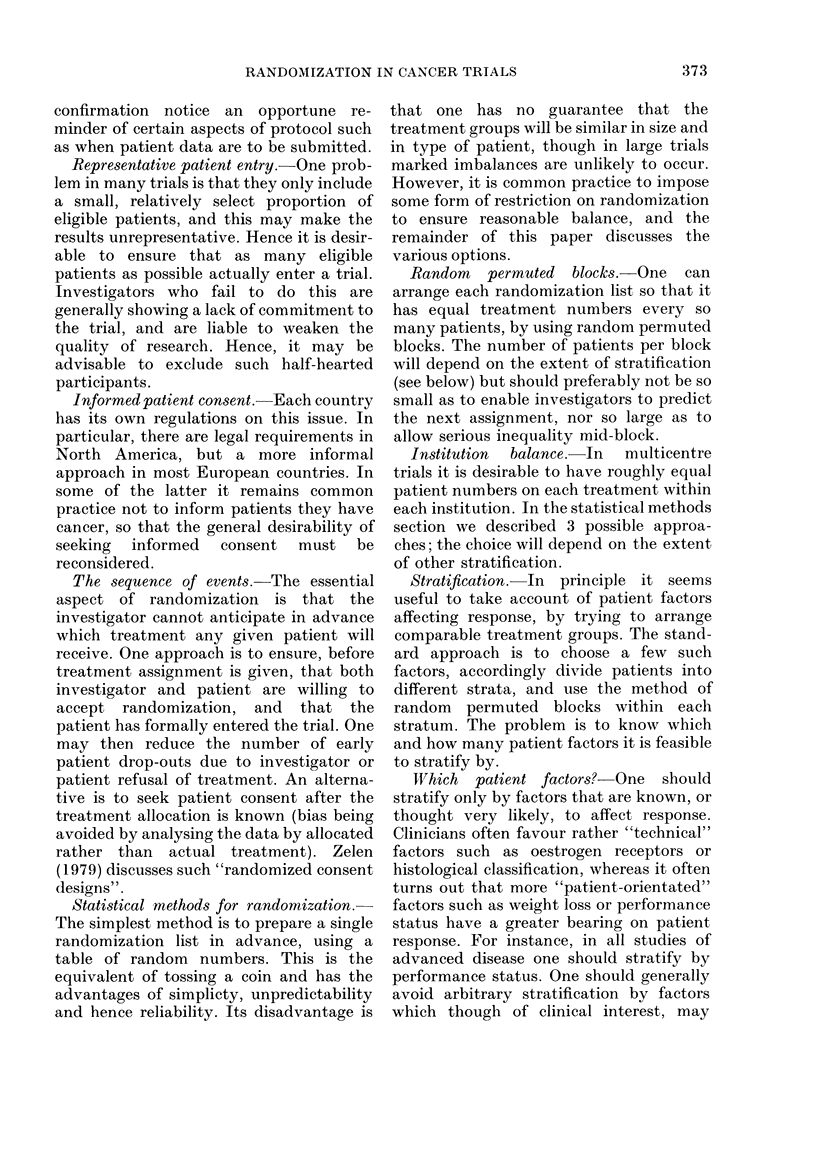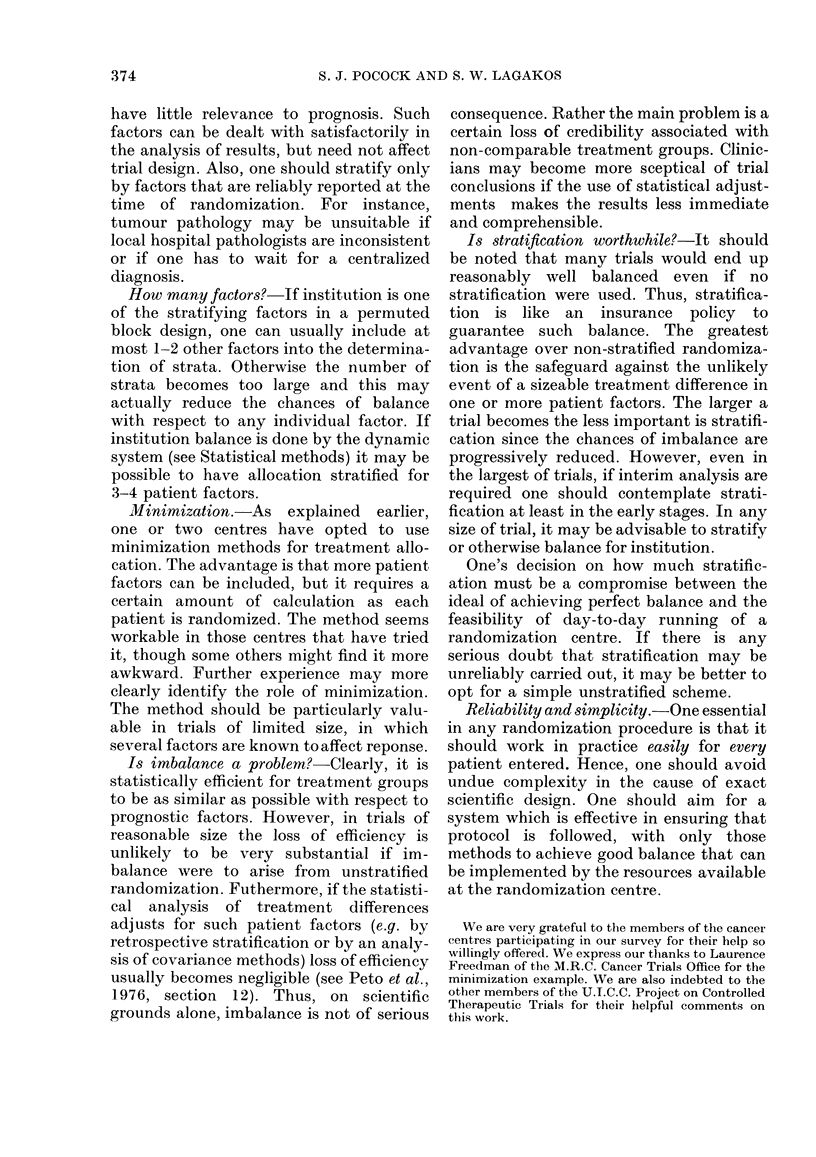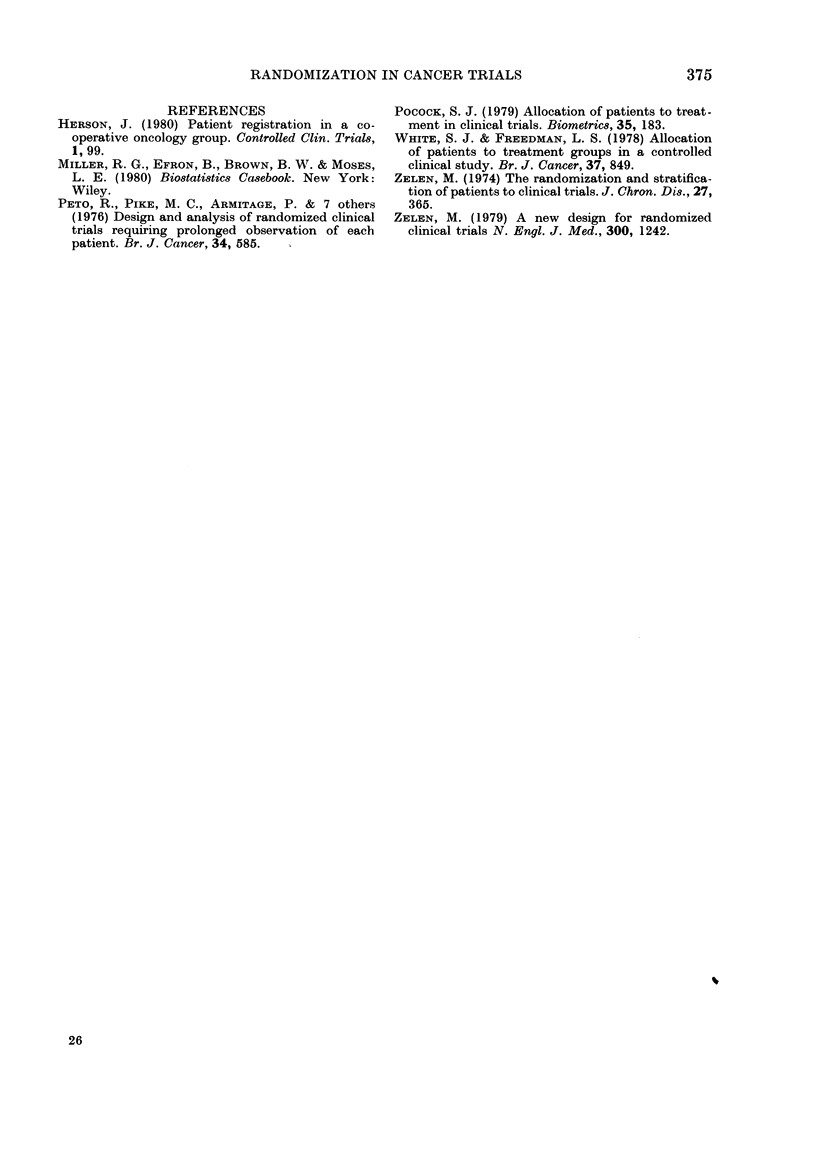# Practical experience of randomization in cancer trials: an international survey.

**DOI:** 10.1038/bjc.1982.212

**Published:** 1982-09

**Authors:** S. J. Pocock, S. W. Lagakos

## Abstract

The results from an international survey of 15 major cancer centres have clarified how randomization is being implemented in cancer trials. As regards the mechanics of obtaining treatment assignment for each patient a system of telephone registration to a central randomization office was widely used. We also advise formal checks for patient eligibility immediately before treatment assignment, and subsequent written confirmation of randomization to the investigators. As regards statistical methods, stratification of randomization by one or two prognostic factors (and institution in multicentre trials) is commonplace. Most centres used the standard approach of random permuted blocks within strata though some others used "dynamic" institution-balancing or "minimization" methods instead. The value of stratified allocation is chiefly for the trial's credibility in having comparable treatment groups, rather than for statistical efficiency. One should avoid overstratification and use only the really important prognostic factors. One essential is that randomization should in practice work for every patient, so undue complexity is to be avoided.


*Br. J. Cancer* (1982) **46**, 368

### PRACTICAL EXPERIENCE OF RANDOMIZATION IN CANCER TRIALS: AN INTERNATIONAL SURVEY

S. J. POCOCK AND S. W. LAGAKOS*

*From the Department of Clinical Epidemiology and General Practice, Royal Free Hospital Medical School, London NW3 and the *Division of Biostatistics and Epidemiology, Sidney Farber Cancer Center, Boston, Ma* 02115, *U.S.A.*

Received 9 February 1982 Accepted 2 April 1982

**Summary.—The results from an international survey of 15 major cancer centres have clarified how randomization is being implemented in cancer trials. As regards the *mechanics* of obtaining treatment assignment for each patient a system of telephone registration to a central randomization office was widely used. We also advise formal checks for patient eligibility immediately before treatment assignment, and subsequent written confirmation of randomization to the investigators. As regards *statistical methods*, stratification of randomization by one or two prognostic factors (and institution in multicentre trials) is commonplace. Most centres used the standard approach of random permuted blocks within strata though some others used "dynamic" institution-balancing or "minimization" methods instead. The value of stratified allocation is chiefly for the trial's credibility in having comparable treatment groups, rather than for statistical efficiency. One should avoid overstratification and use only the really important prognostic factors. One essential is that randomization should in practice work for every patient, so undue complexity is to be avoided.**

THIS ARTICLE has two main objectives: to describe current approaches to implementing randomization by presenting the results of a survey of 15 major cancer trial centres in Europe and the United States, and to make recommendations regarding how randomization should be carried out.

We shall not deal with the controversy over whether studies should be randomized, and so shall not consider any alternatives to randomization. That is, we wish to tackle the question how to randomize, rather than why.

In considering randomization methods it is convenient to consider 2 main topics:

*Mechanics.*—How should treatment assignment actually be obtained for each patient entering a trial? Such issues as assignment by phone or by sealed envelopes, the sequence of events for patient registration, and requisite documentation are considered here.

*Statistical methods.*—What methods should be used to set up the randomization procedure for a trial? For example, are methods other than randomized permuted blocks worth considering, and is stratification necessary?

We recognize that there is no single approach that is suitable for all centres or for all trials. Hence, our recommendations represent a balance between theoretical efficiency and practical feasibility.

### A SURVEY OF CANCER-TRIAL CENTRES

There are several articles describing how randomization and patient registration could be carried out in clinical trials (*e.g.* Herson, 1980; Pocock, 1979; Zelen, 1974). There also exist many more statistical papers which discuss the theoretical properties of various, sometimes very complex, randomization schemes. Our survey was designed to discover the methods by which randomization is actually being performed in cancer centres, why they are used and how well they seem to be working.

We could have directed our enquiries at a random sample of trials or of cancer centres, but this seemed impracticable and not particularly useful. Instead, it seemed more informative to undertake a detailed enquiry at a limited number of established centres which were experienced at coordinating clinical trials. The 15 centres included in our survey are listed below:

| Organization | Location | Contact | |
|---|---|---|---|
| Institut Gustave Roussy | Paris | R. Flamant | Continental Europe |
| Cancer Research Centre of the U.S.S.R. Academy of Medical Sciences | Moscow | A. Klimenkov | |
| European Organization for Research on Treatment of Cancer | Brussels | R. Sylvester | |
| Medical Research Council Cancer Trials Office | Cambridge | L. Freedman | U.K. |
| Christie Hospital | Manchester | M. Palmer | |
| Oxford Clinical Trials Centre | Oxford | R. Peto | |
| Scottish Breast Group | Edinburgh | R. Prescott | |
| Memorial Sloan–Kettering Cancer Center | New York | D. Braun | U.S.A. |
| Northern California Oncology Group | San Francisco | B. Brown | |
| Biometry Branch of National Cancer Institute | Bethesda | D. Byar | |
| Mayo Clinic | Rochester | T. Fleming | |
| University of Texas System Cancer Center | Houston | E. Gehan | |
| Cancer and Leukemia Group B | New York | O. Glidewell | |
| Childrens' Cancer Study Group | Los Angeles | H. Sather | |
| Eastern Cooperative Oncology Group | Boston | K. Stanley | |

The method of enquiry was for one or both of us to visit each centre, or otherwise meet the persons responsible for randomization procedure. In an interview with a formal questionnaire to guide us through, we would enquire about the randomization procedures used. We also obtained, as an example, specific details of one particular trial, usually the most recent primary breast cancer trial, for each centre.

The centres chosen offer a wide geographic coverage in Europe and North America and represent a broad cross-section of cancer trials. Both large multicentre cooperative groups and smaller in-house trials at specific hospitals are included, as well as centres focussing on certain tumour types.

The centres varied considerably in the scale of operation: 8 had $>30$ currently active trials, while another 3 had $<10$. All centres were involved in multicentre trials, though in 3 most of the trials were confined to their own hospital.

*Mechanics of Treatment Assignment*

The great majority of centres (12/15) used a system of *centralized registration by telephone* as the main method of obtaining treatment assignment. The 3 other centres used a system of sealed envelopes; in one this was preferred because of language problems in multicentre trials across several countries. A few of the other centres used sealed envelopes on a limited basis, for their more remote (*e.g.* overseas) institutions. One centre reported "cheating" by an institution which had opened all envelopes in advance.

Three centres had undertaken *double-blind studies* with telephone randomization: 2 had "blinded" drug packages sent in advance to each centre and the appropriate number was given over the phone, whilst the third arranged for the hospital pharmacists to phone for randomization.

*Informed patient consent* before randomization is a legal requirement for all the U.S. trials. One centre reported that the telephone randomization and patient consent were sometimes carried out simultaneously. However, there was no such requirement in the 7 European centres. Two such centres reported that it was up to each local institution to decide on the degree of informed consent that was needed.

In 8 centres a *check on patient eligibility* was undertaken as part of the telephone randomization. This varied from a rather informal general enquiry in some centres to a formal itemized list of checks in others. In one centre, eligibility checks for in-house studies were done in person by the data manager and investigator. With sealed envelopes there is no real scope for eligibility checks.

As regards pretreatment *documentation* it was standard practice to keep a randomization log sheet for each study. For trials with sealed envelopes this relied on investigators notifying the centre. Of the 12 centres using telephone randomization only 4 sent written confirmation of treatment assignment to the institution entering the patient. Centres varied as to what information was asked for over the phone: all required the patient's name, stratifying factors (including the centre which did not use stratified randomization) and institution. Some also required the physician's name, name of person phoning and patient's date of birth.

We asked each centre about any practical problems occurring during randomization and registration of patients. It is difficult to quantify this information, but brief comment seems worthwhile. All centres reported that some *ineligible patients* were randomized into studies, though those centres with formal eligibility checks at randomization reckoned the problem was minor, say $<5\%$. One centre had documented that 6% of randomized patients were later classified as ineligible, this forming part of an annual statistical assessment of what problems arose in each collaborating institution. Another centre reported a rate of 7% known ineligible patients, plus 3% who were considered ineligible because no on-study form was received. Two centres declared that ineligible patients were included in the analysis of results, whilst some others tended to exclude them.

Most centres reported there were no *non-randomized patients* entering their randomized studies. Two centres reported that a few such patients had entered studies which used sealed-envelope randomization, and another recalled a past problem with non-randomized cases. Two centres permitted some non-randomized patients to follow the protocol of randomized studies, though such cases were reported separately.

Centres generally reported only a small proportion of early *patient withdrawals*, though one centre quoted 4% of such cancellations. In general, this problem was mostly related to patient refusal. Most centres included such patients in subsequent trial reports, though two appeared to exclude such refusals from further analysis, which might in principle engender slight bias.

One centre made use of a *computer* during registration and randomization of each patient. The person at the operations office keyed in eligibility and stratification information, while the institution was on the phone, and a computer algorithm based on the "minimization" method described below determined the treatment assignment, which was then conveyed to the phoning institution.

### *Statistical Methods for Randomization*

For the 15 centres in our survey the most common approach was to produce *randomization lists* before the trial started, using the method of *random permuted blocks within strata*. This means that patients were categorized into several different strata based on specific patient prognostic factors. A separate list of random treatment assignments was then produced for each patient type, making sure that each such list had equal numbers on each treatment every so many patients. This general approach was standard practice in 13/15 centres, though there was considerable variation over details.

First, consider the issue of *stratification*. How many patient factors (other than their institution) were used to form strata? This is hard to quantify, since trials involve different numbers of patients and treatment groups. However, it is informative to list the separate reports from each centre as follows:

| No. of stratifying factors (other than institution) | No. of strata | |
|---|---|---|
| 0 | 1 | European centres |
| 0, except for one trial | 1 | |
| 0–1 | 1–2 | |
| 0–2 | 1–4 | |
| 1 | 2–4 | |
| 1, or occasionally 2 | 2–4 | |
| 1–2 | 2–4 | |
| Typically 3 | Typically 8 | U.S. centres |
| 2–3 | $\geqslant 4$ | |
| 2–4 | Typically 8 | |
| 2–5 | 4–50 | |
| Typically 2 | 4–6 | |
| $\leqslant 3$ | $\leqslant 8$ | |

Clearly, there is great diversity in the amount of stratification used. The American centres were generally more in favour of stratification than the European centres. Indeed 2 European centres generally did not stratify by any factor other than institution, and 2 more had some trials without stratification. No centre had used more than 5 patient factors for random permuted blocks within strata.

Some form of *institution balancing*, to ensure that similar treatment numbers occur within each institution, was standard practice for all except one of the centres with multicentre trials. This was usually done by having institution as another stratifying factor, the only such factor in 2 centres. Two other centres adopted a *dynamic system*: if the treatment difference within the next patient's institution exceeded a certain number (say 2 or 3) the conventional stratified random assignment was interrupted and the treatment with the smallest number in that institution was assigned to that patient. Two other centres included institution as another factor in the minimization method described below.

The most common choice of *size of random permuted blocks* within each stratum was twice the number of treatments. One centre was more restrictive, using blocks of 2 for 2-treatment trials. No centre used blocks of more than 10 patients. One centre used blocks of 2–4 patients in small trials and 6–8 patients in larger trials. Another centre used block sizes from 1–4 times the number of treatments, varied at random within each trial.

Seven centres used a computer program to generate the randomization lists before initiating a trial, whilst others used tables of random numbers.

Four centres had used another rather different method, sometimes called "minimization", for achieving stratified randomization. One of these centres always used this method, another in 80% of trials and the other 2 in one specific trial. This method may be best illustrated by an example from one centre, a head-and-neck trial with 3 relevant factors: 15 institutions, 7 disease sites and nodal involvement (+ or −). For conventional stratification this leaves $15 \times 7 \times 2 = 210$ strata, which is clearly impractical for random permuted blocks. The minimization method attempts to balance dynamically the treatment groups each time a patient is entered into the study. One checks for each possible treatment assignment, what would be the resultant difference in treatment numbers within his institution, his disease site and his nodal involvement category. One then uses these differences to define an imbalance score for that treatment (*e.g.* the sums of squared differences is often used, and is equivalent to the simple calculation given below). One assigns the treatment with the smallest imbalance score.

For instance, suppose 260 patients had entered the above-mentioned head-and-neck cancer trial, and that the next patient was from the Royal Marsden Hospital, Sutton, had cancer of the oropharynx and no nodal involvement. The numbers of patients on each treatment in each of these 3 categories were as follows:

| | Misonidazole | Placebo |
|---|---|---|
| No nodal involvement | 80 | 80 |
| Cancer of oropharynx | 25 | 23 |
| Royal Marsden Hospital | 13 | 14 |
| Sum = | 118 | 117 |

This column total is smaller on placebo, in which case placebo is assigned to that next patient. If the sums were equal, treatment is assigned at random. There are various possible elaborations on minimization, which become feasible if a computer is available at the time of randomization; the above example has the advantage of simplicity. Implementation requires a current record of numbers of patients on each treatment for each level of each factor, *i.e.* $(15+7+2) \times 2 = 48$ numbers, of which 6 are used at any one assignment. In practice, the necessary calculation can be done while the investigator is on the phone waiting for the randomized assignment.

In using minimization there is no particular restrictive limit on the number of stratifying factors; *e.g.* a trial for lung cancer at another centre stratified by age, extent of disease, histology, sex, performance status and institution. White & Freedman (1978) and Miller *et al.* (1980) give further details of minimization and other statistical methods.

The use of *unequal randomization*, *i.e.* more patients on one treatment than another, was not very common, though 7 centres had tried it. The future of unequal randomization will depend on the experiences in recent trials where it has been used.

We have so far discussed stratification in general terms. One key specific issue is deciding *which patient factors* to stratify by. To illustrate this problem we focus on trials for primary breast cancer. Ten centres had an active primary breast trial and the factors (other than institution) which they used for stratification are listed below (centre numbers do not correspond to the earlier list of centres).

| Centre | Stratification factors |
|---|---|
| 1 | None |
| 2 | None |
| 3 | Stages I or II<br>Pre- or post-menopausal |
| 4 | +ve nodes (+ or −)<br>Radiotherapy (+ or −) |
| 5 | No. +ve nodes<br>Pre- or post-menopausal |
| 6 | Simple or radical mastectomy<br>Pre- or post-menopausal |
| 7 | No. +ve nodes, (1–3 or 4+)<br>Pre- or post-menopausal<br>Oestrogen receptor, (+ or −) |
| 8 | No. +ve nodes, (0–3 or 4+)<br>Tumour size, ( $< 3$ or $\geqslant 3$ cm)<br>Pre- or post-menopausal<br>Unfavourable signs (+ or −) |
| 9 | No. +ve nodes, (1–3 or 4+)<br>Oestrogen receptor, (+ or −) |
| 10 | No. +ve nodes (1–3 or 4+)<br>Pre- or post-menopausal<br>Tumour size ( $< 2$, 2–5 or $> 5$ cm)<br>Radiotherapy (+ or −)<br>Oestrogen receptor, (+ or −) |

Evidently a variety of choices has been made. Two trials had no factors (+ or −) while another had 5 factors, with a total of 48 strata. One can see some consistency in the choice of factors, no. +ve nodes and menopausal status being fairly standard. Some of the trials were without these patient factors, because they were restricted to node −ve or pre-menopausal patients. Perhaps the reliability and value of oestrogen receptor status for stratifying might be questioned. The inclusion of tumour size as a stratifying factor appears to depend on the number of strata a centre considers appropriate and manageable.

### RECOMMENDATIONS

Having reviewed how various cancer centres handle patient registration and randomization, we will make some general recommendations:

*Telephone randomization.*—For multicentre trials, the most reliable means of randomizing patients is to have the investigator telephone a coordinating centre each time he enters a patient. The alternative of using sealed envelopes at each institution does not allow central monitoring of randomization procedure, and carries a greater risk of things going wrong. If cental randomization is not feasible (*e.g.* in some international trials) sealed envelopes may have to be used. It is then important to monitor patient entry retrospectively to check that the scheme was followed. For single-centre trials, one should aim for the same formal approach *via* a randomization office. If, however, personal contact then replaces the telephone call, formal procedures may be necessary to prevent the next treatment being known before the final decision is made to admit a particular patient.

*Eligibility checks.*—Some centres undertook a formal check that each patient was eligible for the trial immediately before treatment was assigned. We endorse this approach as an effective method of reducing the number of patients mistakenly put on to a trial. While one can try to preserve a trial's validity by retrospectively eliminating ineligible cases, suspicions may arise if it occurs frequently. More importantly, the inclusion of ineligible patients in a study may mean that they fail to receive the best treatment for their disease. Whether to include or exclude ineligible patients in the analysis of trial results should ideally be stated in the study protocol, so as to avoid any biased decisions later.

*Confirmation of randomization.*—In multicentre trials, it is advisable to follow up telephone randomization, by sending the investigator a written confirmation of patient entry and treatment assignment. This was only done in a few centres, and one wonders what drops-outs might occur in other centres due to lack of confirmation. One can also include with the confirmation notice an opportune reminder of certain aspects of protocol such as when patient data are to be submitted.

*Representative patient entry.*—One problem in many trials is that they only include a small, relatively select proportion of eligible patients, and this may make the results unrepresentative. Hence it is desirable to ensure that as many eligible patients as possible actually enter a trial. Investigators who fail to do this are generally showing a lack of commitment to the trial, and are liable to weaken the quality of research. Hence, it may be advisable to exclude such half-hearted participants.

*Informed patient consent.*—Each country has its own regulations on this issue. In particular, there are legal requirements in North America, but a more informal approach in most European countries. In some of the latter it remains common practice not to inform patients they have cancer, so that the general desirability of seeking informed consent must be reconsidered.

*The sequence of events.*—The essential aspect of randomization is that the investigator cannot anticipate in advance which treatment any given patient will receive. One approach is to ensure, before treatment assignment is given, that both investigator and patient are willing to accept randomization, and that the patient has formally entered the trial. One may then reduce the number of early patient drop-outs due to investigator or patient refusal of treatment. An alternative is to seek patient consent after the treatment allocation is known (bias being avoided by analysing the data by allocated rather than actual treatment). Zelen (1979) discusses such "randomized consent designs".

*Statistical methods for randomization.*—The simplest method is to prepare a single randomization list in advance, using a table of random numbers. This is the equivalent of tossing a coin and has the advantages of simplicty, unpredictability and hence reliability. Its disadvantage is that one has no guarantee that the treatment groups will be similar in size and in type of patient, though in large trials marked imbalances are unlikely to occur. However, it is common practice to impose some form of restriction on randomization to ensure reasonable balance, and the remainder of this paper discusses the various options.

*Random permuted blocks.*—One can arrange each randomization list so that it has equal treatment numbers every so many patients, by using random permuted blocks. The number of patients per block will depend on the extent of stratification (see below) but should preferably not be so small as to enable investigators to predict the next assignment, nor so large as to allow serious inequality mid-block.

*Institution balance.*—In multicentre trials it is desirable to have roughly equal patient numbers on each treatment within each institution. In the statistical methods section we described 3 possible approaches; the choice will depend on the extent of other stratification.

*Stratification.*—In principle it seems useful to take account of patient factors affecting response, by trying to arrange comparable treatment groups. The standard approach is to choose a few such factors, accordingly divide patients into different strata, and use the method of random permuted blocks within each stratum. The problem is to know which and how many patient factors it is feasible to stratify by.

*Which patient factors?*—One should stratify only by factors that are known, or thought very likely, to affect response. Clinicians often favour rather "technical" factors such as oestrogen receptors or histological classification, whereas it often turns out that more "patient-orientated" factors such as weight loss or performance status have a greater bearing on patient response. For instance, in all studies of advanced disease one should stratify by performance status. One should generally avoid arbitrary stratification by factors which though of clinical interest, may have little relevance to prognosis. Such factors can be dealt with satisfactorily in the analysis of results, but need not affect trial design. Also, one should stratify only by factors that are reliably reported at the time of randomization. For instance, tumour pathology may be unsuitable if local hospital pathologists are inconsistent or if one has to wait for a centralized diagnosis.

*How many factors?*—If institution is one of the stratifying factors in a permuted block design, one can usually include at most 1–2 other factors into the determination of strata. Otherwise the number of strata becomes too large and this may actually reduce the chances of balance with respect to any individual factor. If institution balance is done by the dynamic system (see Statistical methods) it may be possible to have allocation stratified for 3–4 patient factors.

*Minimization.*—As explained earlier, one or two centres have opted to use minimization methods for treatment allocation. The advantage is that more patient factors can be included, but it requires a certain amount of calculation as each patient is randomized. The method seems workable in those centres that have tried it, though some others might find it more awkward. Further experience may more clearly identify the role of minimization. The method should be particularly valuable in trials of limited size, in which several factors are known to affect reponse.

*Is imbalance a problem?*—Clearly, it is statistically efficient for treatment groups to be as similar as possible with respect to prognostic factors. However, in trials of reasonable size the loss of efficiency is unlikely to be very substantial if imbalance were to arise from unstratified randomization. Futhermore, if the statistical analysis of treatment differences adjusts for such patient factors (*e.g.* by retrospective stratification or by an analysis of covariance methods) loss of efficiency usually becomes negligible (see Peto *et al.*, 1976, section 12). Thus, on scientific grounds alone, imbalance is not of serious consequence. Rather the main problem is a certain loss of credibility associated with non-comparable treatment groups. Clinicians may become more sceptical of trial conclusions if the use of statistical adjustments makes the results less immediate and comprehensible.

*Is stratification worthwhile?*—It should be noted that many trials would end up reasonably well balanced even if no stratification were used. Thus, stratification is like an insurance policy to guarantee such balance. The greatest advantage over non-stratified randomization is the safeguard against the unlikely event of a sizeable treatment difference in one or more patient factors. The larger a trial becomes the less important is stratification since the chances of imbalance are progressively reduced. However, even in the largest of trials, if interim analysis are required one should contemplate stratification at least in the early stages. In any size of trial, it may be advisable to stratify or otherwise balance for institution.

One's decision on how much stratification must be a compromise between the ideal of achieving perfect balance and the feasibility of day-to-day running of a randomization centre. If there is any serious doubt that stratification may be unreliably carried out, it may be better to opt for a simple unstratified scheme.

*Reliability and simplicity.*—One essential in any randomization procedure is that it should work in practice *easily* for *every* patient entered. Hence, one should avoid undue complexity in the cause of exact scientific design. One should aim for a system which is effective in ensuring that protocol is followed, with only those methods to achieve good balance that can be implemented by the resources available at the randomization centre.

We are very grateful to the members of the cancer centres participating in our survey for their help so willingly offered. We express our thanks to Laurence Freedman of the M.R.C. Cancer Trials Office for the minimization example. We are also indebted to the other members of the U.I.C.C. Project on Controlled Therapeutic Trials for their helpful comments on this work.

### REFERENCES

HERSON, J. (1980) Patient registration in a co-operative oncology group. *Controlled Clin. Trials*, **1**, 99.

MILLER, R. G., EFRON, B., BROWN, B. W. & MOSES, L. E. (1980) *Biostatistics Casebook*. New York: Wiley.

PETO, R., PIKE, M. C., ARMITAGE, P. & 7 others (1976) Design and analysis of randomized clinical trials requiring prolonged observation of each patient. *Br. J. Cancer*, **34**, 585.

POCOCK, S. J. (1979) Allocation of patients to treatment in clinical trials. *Biometrics*, **35**, 183.

WHITE, S. J. & FREEDMAN, L. S. (1978) Allocation of patients to treatment groups in a controlled clinical study. *Br. J. Cancer*, **37**, 849.

ZELEN, M. (1974) The randomization and stratification of patients to clinical trials. *J. Chron. Dis.*, **27**, 365.

ZELEN, M. (1979) A new design for randomized clinical trials *N. Engl. J. Med.*, **300**, 1242.